# Oxidative stress concept updated: Definitions, classifications, and regulatory pathways implicated

**DOI:** 10.17179/excli2021-3596

**Published:** 2021-05-26

**Authors:** Volodymyr I. Lushchak, Kenneth B. Storey

**Affiliations:** 1 Department of Biochemistry and Biotechnology, Vasyl Stefanyk Precarpathian National University, 57 Shevchenko Str., Ivano-Frankivsk, 76018, Ukraine; 2 I. Horbachevsky Ternopil National Medical University, 1 m.Voli, Ternopil, 46002, Ukraine; 3 Research and Development University, 13a Shota Rustaveli Str., Ivano-Frankivsk, 76018, Ukraine; 4 Department of Biology, Carleton University, 1125 Colonel By Drive, Ottawa, Ontario K1S 5B6, Canada

**Keywords:** adaptation, cysteine residues, Nrf2, OxyR, reversible oxidation, SoxRS, Yap1

## Abstract

Reactive oxygen species were discovered in living organisms in the early 1950's and their action has been implicated in diverse biological processes. First formulated by H. Sies in 1985[57], the oxidative stress concept stimulated substantial interest in reactive oxygen species and it is now common that fundamental research in various biomedical fields includes mention of research on the involvement of oxidative stress. Such strong interest has resulted in the development of definitions and classifications of oxidative stress and much research progress in the field. Although we clearly understand the limitations of various definitions or classifications, such parameters may help to provide quantitative descriptions, compare related processes among different laboratories, and introduce some measurable parameters. This paper highlights recent advances in the areas of oxidative stress definitions and the classification of oxidative stresses. Such items are directly associated with our understanding of the molecular mechanisms involved in organismal responses to oxidative insults. The knowledge accumulated to date indicates that selective expression of specific genes is a central player in the adaptive response to oxidative stress and reversible oxidation of cysteine residues of sensor proteins is a key process regulating responses to oxidative stress.

## Introduction

Free radicals (FR) were discovered at the beginning of the 20^th^ century by Moses Gomberg (1900[23]). In the 1930's, Leonor Michaelis proposed that FR mediated most, if not all, oxidation reactions involving organic molecules (Michaelis, 1939[43]). Although this statement was generally wrong, it stimulated substantial interest in FR participation in different chemical transformations. Initially, it was believed that the high chemical reactivity of FR was not compatible with their presence in living organisms. However, in the early 1950's FR were found in biological systems (Commoner et al., 1954[10]) and virtually immediately were proposed to be implicated in diverse pathologies (Gerschman et al., 1954[21]) and aging (Harman, 1956[25]). Many of these predictions proved to be correct and we now have substantial information supporting FR involvement in pathologies and aging. The free radical theory of aging is probably the best supported experimentally and is well appreciated by the scientific community (Harman, 2003[27], 2006[26]; Viña et al., 2013[63]; Gladyshev, 2014[22]). 

The concept of oxidative stress was introduced by Helmut Sies in 1985 in an introductory chapter 1 of a book entitled “Oxidative Stress” where he also gave the first definition of this phenomenon (Sies, 1985[57]). This seminal work and others by H. Sies inspired many investigations in the field of oxidative stress such that, at present, the oxidative stress concept is well accepted and extensively used in both basic and applied fields of biology and medicine (Storey, 1996[61]; Freire et al., 2011[18]; Jones and Sies, 2015[30]; Cadenas et al., 2016[6]; Salim, 2017[51]; Islam, 2017[29]; Garaschuk et al., 2018[19]; Lushchak, 2021[39]). Furthermore, it is an important concept in the adaptation to environmental stress by organisms (Storey, 1996[61]; Lushchak, 2011[34][36]). Therefore, to provide further progress in the field of oxidative stress there is a need to update several points and shed light on them. In this paper, we focus on three aspects of the oxidative stress concept: definitions, classifications, and regulatory pathways involved in the normal (adequate) adaptive response to oxidative stress. Diverse pathologies are outside the scope of this paper. 

## Definitions

As mentioned above, the first definition of oxidative stress was given by H. Sies in 1985[57]: “a disturbance in the prooxidant-antioxidant balance in favor of the former, leading to potential damage”. The disturbed balance between pro- and antioxidants is a key point in this definition. Later the definition was updated by the author to “An imbalance between oxidants and antioxidants in favour of the oxidants, potentially leading to damage, is termed “oxidative stress”” (Sies, 1997[58]). Two aspects have to be noted here: (1) since FRs are very short-lived species, they cannot be accumulated and, therefore, one should speak only about their steady-state levels; and (2) research has shown that FR are not only deleterious species but are involved in a number of normal regulatory processes. In order to reflect such developments, one of us proposed that a somewhat altered definition of oxidative stress should be: “Oxidative stress is a situation when steady-state ROS concentration is transiently or chronically elevated, disturbing cellular metabolism and its regulation and damaging cellular constituents” (Lushchak, 2011[36]). Finally, probably the best definition of oxidative stress at the present time may be: “Oxidative stress is a transient or long-term increase of steady­state ROS levels, disturbing cellular metabolic and signaling pathways, particularly ROS­based ones, and leading to oxidative modifications of an organism's macromolecules that, if not counterbalanced, may culminate in cell death via necrosis or apoptosis”. 

Two important points must be mentioned here. The field of oxidative stress is full of misunderstandings of terms. Therefore, we point out that the group of reactive oxygen species (ROS) mainly encompasses superoxide anion radical (O_2_^•^^−^), hydrogen peroxide (Н_2_О_2_) and hydroxyl radical (HO^•^). Hydrogen peroxide is not a radical, whereas O_2_^•^^− ^and HO^•^ are free radicals. In order to avoid misunderstanding in this paper, we allow the term ROS to include hydrogen peroxide (interested readers can read more about this in previous work) (Lushchak, 2014[37]). It also must be mentioned that intracellular ROS homeostasis is generally very tightly controlled providing extremely low ROS levels. In eukaryotes, intracellular hydrogen peroxide was first identified in perfused liver cells by H. Sies and B. Chance (1970[60]). This pioneering work provided the first methodological tool to measure hydrogen peroxide levels in cells. In diverse organisms and cells, intracellular hydrogen peroxide concentrations were found to range from 10 to 100 nM (Chance et al., 1979[7]). Later, in MCF-7 cells, Н_2_О_2 _intracellular concentrations were measured at 2.5-12.5 µM (de Oliveira-Marques et al., 2007[11]). More recent quantification of intracellular hydrogen peroxide levels showed that in cultured HeLa cells normal Н_2_О_2 _concentrations were less than 7.7 nM whereas higher values were induced by some pathological deviations (Huang and Sikes, 2014[28]), thus correlating well with older data (Chance et al., 1979[7]). 

## Classifications

In living organisms, ROS monitoring is a very complicated task and there is actually no reliable technique to quantitatively and directly evaluate ROS levels either *in vitro* or *in vivo* due to their inherent reactivity (Arteel, 2016[2]). These species are multifaceted and, because of this, different parameters may be used for their characterization. In practice, scientists have to use indirect detection of “footprints” of ROS-related processes which results in a very large “black box” of uncertainty. Currently there is no way to investigate ROS processes directly in an accurate manner. Indeed, we are still only at the beginning of understanding the myriad of vital processes in which ROS are involved. Such complexity of ROS-related processes also leaves us with no good basis for classification of the diverse forms of oxidative stress. Hence, understanding the limitations of potential classifications, we have launched the first attempt to create a definitive classification that may stimulate interest in the field. Two of such classification systems have been proposed: (1) time based (temporal), and (2) concentration/intensity based.

### Time-course based classification

**Figure 1:**
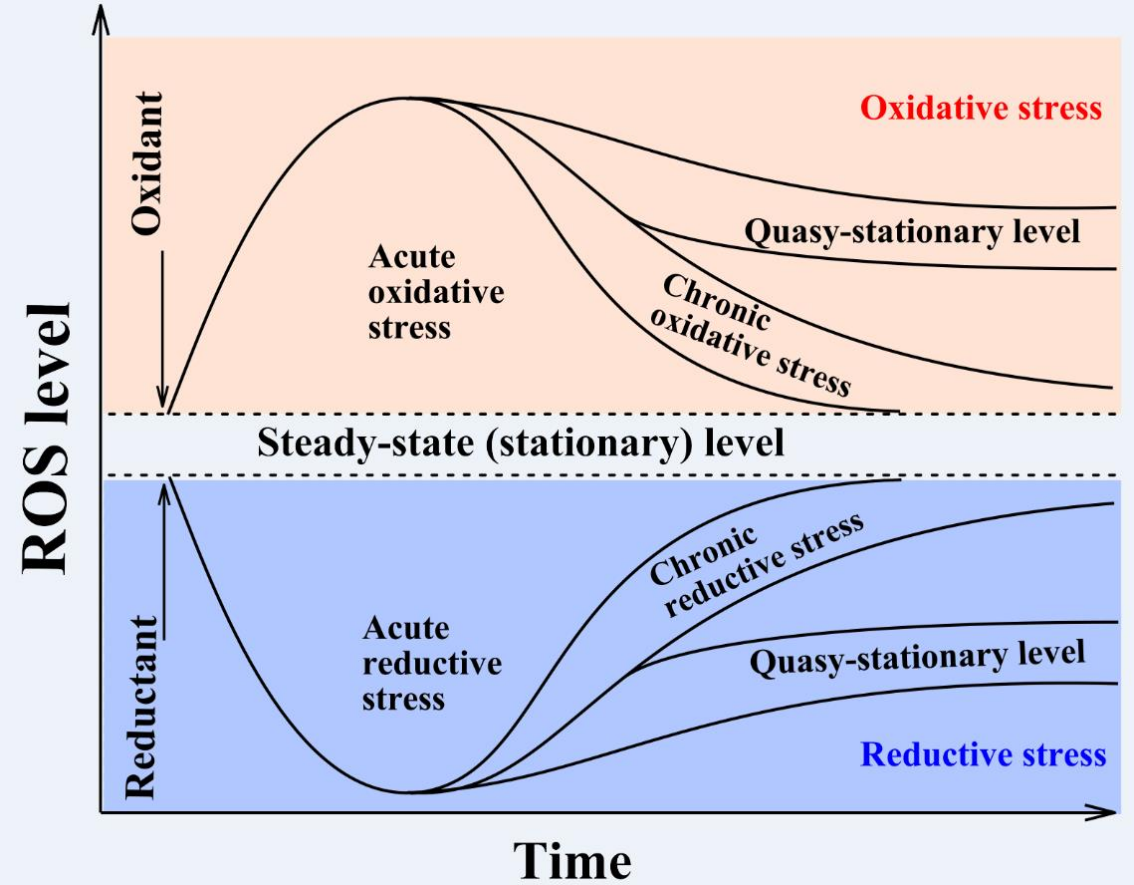
Schematic presentation of the time-course based classification of oxidative stresses. Usually a steady-state ROS concentration can be maintained within a certain range and fluctuates like other parameters in the body according to homeostasis theory. However, under certain circumstances, ROS concentration may exit from this corridor due to increased generation or decreased elimination of ROS. The situation when ROS levels increase for a short time period with certain functional consequences is called “acute oxidative stress”, whereas a prolonged increase in ROS levels accompanied by such consequences is called “chronic oxidative stress.” In some cases, ROS levels do not return into the original corridor or stabilize close to it and or even stabilize at a higher steady-state level, called a quasi-stationary state. Both acute and chronic oxidative stresses may affect living organisms differently and cause more or less significant damage to cells and, if the system is unable to regain control, can lead to cell death by apoptosis or necrosis. The reverse situation when steady-state ROS concentrations decrease relative to the initial level has been called “reductive stress” (modified from Lushchak, 2014b, under the CC BY 4.0 license).

Under normal conditions, ROS levels fluctuate within a certain range called the steady-state level (Figure 1(Fig. 1)). With a change of conditions (e.g. application of an external oxidant), ROS levels may increase. If efficiency of antioxidant defense is high enough, ROS levels may return into the initial range within minutes. Such stress has been called “acute oxidative stress”. It is supposed that the response to acute oxidative stress does not require stimulation of gene expression to neutralize ROS. However, in many cases living systems cannot cope easily/quickly with even a single application of an oxidative stress inducer due to which enhanced ROS levels are maintained for a longer time such as for hours, days or more. This stress has been called “chronic oxidative stress” and usually involves expression of various stress-associated genes. There are at least two types of chronic oxidative stress: (1) when ROS levels are either extended or slightly increased out of the normal steady-state ROS range and are reliably higher than the initial state, or (2) ROS levels are stabilized at a new higher so-called quasi-stationary level. In our opinion, the latter changes may be connected with a new physiological state of the organism. 

It should be added that virtually the same logic may be applied in the reverse situation when ROS level is decreased (Figure 1(Fig. 1)). Such a situation has been called “reductive stress” (Lushchak, 2011[34]) and may be accompanied by increased levels of reduced glutathione and redox coenzymes like NAD(P)H. Since the concept of reductive stress is not well developed even from a methodological point of view, we just mention it here and will not analyze it further. However, it is clear that both stresses can be combined in the term “redox stress”. This could include perturbation of redox homeostasis under stressful conditions. This approach is well supported by the concept of “The Redox Code” proposed by Jones and Sies (2015[30]). 

**Figure 2:**
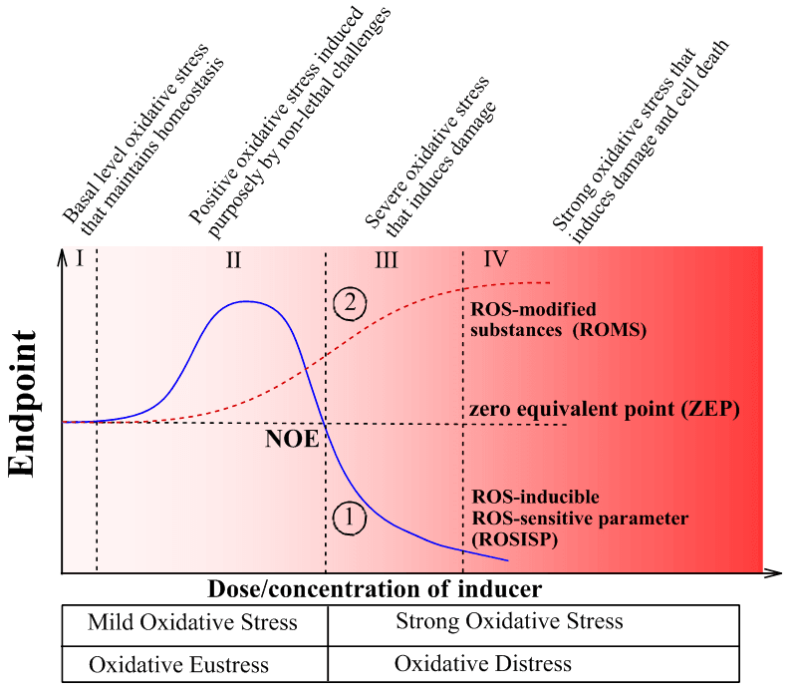
Schematic presentation of the intensity-based classification of oxidative stresses. Curve 1 shows the path of a ROS-induced ROS-sensitive parameter (ROSISP), for example, the activity of an antioxidant enzyme, whereas curve 2 indicates the path of a ROS-modified cellular substance (ROMS), for example, oxidized lipids, proteins or nucleic acids. In fact, curves 1 and 2 show the relationship between the dose/concentration of the oxidative stress inducer and parameters typically used to characterize the stress and that can be experimentally measured. Region I - basal oxidative stress (BOS) is where there are no observable effects due to a very low intensity of oxidative stress; Region II - low intensity oxidative stress (LOS) with a slightly increased level of ROS-modified molecules and enhanced activity of antioxidant enzymes in response to oxidative stress; Region III - strong oxidative stress (SOS); and Region IV - very strong oxidative stress (VOS), where the values ​​of the recorded parameters reach nearly maximum/minimum values. Abbreviations: NOE - no observable effect point; ZEP - zero equivalent point where the levels of components of interest correspond to the initial (basic) level in the absence of an inducer of oxidative stress. (Modified from Lushchak, 2014b, under the CC BY 4.0 license).

Intensity-based oxidative stress classification connects the dose/intensity/concentration of the inducer with the biological responses of the system (Figure 2(Fig. 2)). In this case, it does not matter what induces oxidative stress: physical or chemical factors or their combination. It is the result that matters: an increased ROS steady-state level. Two measurable parameters of living organisms are important to assess to get quantitative parameters for intensity-based classifications: (1) ROS-inducible ROS-sensitive parameters (ROSISP, Figure 2, curve 1(Fig. 2)) and (2) ROS-modified substances (ROMS, Figure 2, curve 2(Fig. 2)). The first group, ROSISP, includes the activities of antioxidant enzymes. These may also be modified by ROS (e.g. inactivated) or they may be up-regulated by ROS, depending on the situation. The second group, ROMS, includes any cellular/extracellular product resulting from an interaction between ROS and any component of a living organism. The only important requirement for analyzing of ROMS is a reliable method for measuring ROMS levels and the relative stability of the ROMS molecule(s) in order to provide good reproducibility of the measurements. The ROMS parameters used for classification may include ROS-modified lipids, proteins, carbohydrates and/or nucleic acids (Lushchak, 2014[37][35]). 

It is well established that antioxidant enzymes can be inactivated by ROS (Semchyshyn et al., 1999[55]; Bayliak et al., 2006[4]; Gottfredsen et al., 2013[24]; Riethmüller et al., 2015[50]). At the same time, a moderate increase in ROS levels can up-regulate antioxidant enzyme levels in all phylogenetic groups assessed to date (Lushchak, 2011[34]). The mechanisms involved in such up-regulation are discussed below and here we just note that the ROSISP (Figure 2, curve 1(Fig. 2)) is a superposition of two processes - ROS-induced inactivation and ROS-induced up-regulation. Since the sensitivity of both processes to ROS levels is different, the integrative response of the systems to the variation of ROS concentration is very difficult to predict. Let us try to analyze effects of ROS concentrations (also maybe dose of inducer) on ROSISP and ROMS.

At low doses/concentrations of an externally added inducer neither ROSISP nor ROMS differ from the control values and, despite an intuitively expected induction of oxidative stress, this cannot be proven quantitatively due to limitations of the methods applied that often cannot register very small changes in the parameters measured. Therefore, stress in this region has been called “basal oxidative stress” (BOS) (Figure 2, region I(Fig. 2)). Exposure to higher doses/concentrations of the inducer results in recordable changes in the levels of ROMS and ROSISP (region II). However, since ROMS gradually increase in region II this is called “low intensity oxidative stress” (LOS). ROSISP respond in a two-phase manner: in the first phase they increase, but after peaking at a certain ROS value they decrease in a second phase to a so-called zero equivalent point (ZEP) where no observed effect (NOE) is seen. Biphasic behavior of ROSISP in region II may be explained by the prevailing up-regulation in the first phase, which to some extent masks ROS-promoted inactivation and, during the second phase, up-regulation is minimized due to extensive inactivation by the inducer, finally resulting in lower ROSISP. The next region III has been called “strong oxidative stress” (SOS) where ROSISP is clearly lower than initial or region I values, whereas ROMS is higher than in region III. Finally, at maximal dose/concentrations of the inducer there is a region IV called “very strong oxidative stress” (VOS). Here ROSISP reaches minimum values due to maximal ROS-induced inactivation and in some cases ROSISP may reach an asymptote or zero value, whereas ROMS reaches maximum values due to exhaustion of cellular substrates that can be modified by ROS. 

Recently H. Sies (2017[56]) adapted and generalized elements of H. Selye's Stress Theory (Selye, 1974[52]) and defined “oxidative eustress” as “physiological oxidative stress” to discriminate it from “oxidative distress” with excessive loads causing oxidative damage. Such differentiation corresponds rather well to our earlier proposal that simplified the intensity-based classification of oxidative stresses (Lushchak, 2014[37]). In this case “oxidative eustress” corresponds to “mild oxidative stress”, and “oxidative distress” to “severe (strong) oxidative stress”. These relationships are depicted at Figure 2(Fig. 2). 

### Intensity-based classification

It was proposed by one of us (Lushchak, 2014[35]) that, depending on its intensity, oxidative stress may be classified as basal, low intensity, intermediate intensity and high intensity oxidative stress. Unfortunately, to date, no experimental support for this theoretical concept has been developed. The main problem is that the markers of oxidative stress routinely used for evaluation of its intensity show different sensitivities to the effects of the inducer due to which the responses of an organism can be categorized as different types of stress. 

## Regulatory Pathways Involved

Since the first definition of oxidative stress (Sies, 1985[57]), molecular mechanisms of ROS-induced up-regulation of antioxidant enzymes have been discovered. At least two features of such up-regulation have to be noted: (1) ROS-promoted up-regulation is mainly regulated at the gene/protein expression level, and (2) reversible oxidation of sensor thiol groups is, in most cases, responsible for the initiation of ROS-sensitive regulatory cascades. The first statement clearly indicates that despite the stress and enhanced energy demands to cope with oxidative stress in order to overwhelm its negative effects, organisms reallocate resources to defense systems to prevent ROS-induced injury. Such events necessitate differential expression of selected genes encoding antioxidant and associated enzymes whereas expression of most other genes may be suppressed while attention is given to dealing with oxidative stress. Since these situations have been described many times (Storey, 1996[61]; Lushchak, 2011[34][36], 2014[37]; Picone et al., 2015[48]; Lichtenberg and Pinchuk, 2015[31]; El-Terras et al., 2016[14]; Förster and Reiser, 2016[17]; Done and Traustadóttir, 2016[13]), we do not cover them in this paper. 

**Figure 3:**
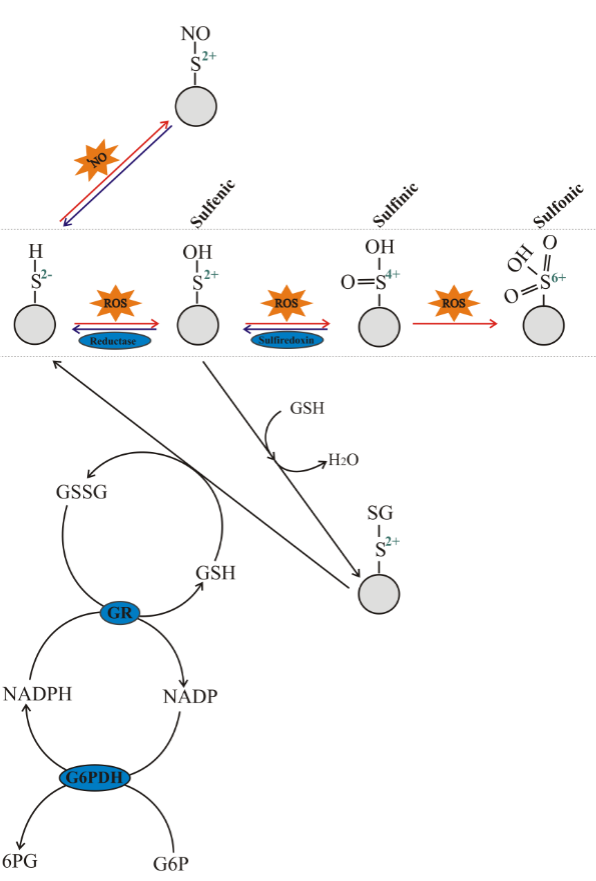
Oxidative pattern of cysteine residues in proteins: sulfenic, sulfinic, or sulfonic derivatives and the possibilities for their reduction. In biological systems, organosulfur sulfenic and sulfinic derivatives may be reduced by thioredoxin and sulfiredoxin, respectively, whereas the sulfonic form is not reduced by these agents. Glutathionylated proteins are formed by direct interaction of GSH with sulfenic acid derivatives, exchange between cysteine residues and GSSG, or interaction with oxidized glutathione forms. Formation of S-nitrosothiols, containing a nitroso group attached to the sulfur atom of a thiol, may be the way of protecting thiol groups against oxidation and stabilization or transportation of unstable nitric oxide. Abbreviations: G6P - glucose-6-phosphate; 6PG - 6-phosphogluconate; GR - glutathione reductase; G6PDH - glucose-6-phosphate dehydrogenase

There are at least four defense strategies of protection against ROS: prevention, interception, repair and elimination of ROS-modified molecules (Sies, 1993[59]; Lushchak, 2014[37]). Of these, interception via regulation of enzymatic antioxidant systems has caught the most attention in the field. The molecular mechanisms of adaptive responses to oxidative stress were first described in bacteria. In these organisms, there are at least two systems differing in their basic molecular mechanisms of sensing redox signals from ROS, namely SoxRS and OxyR (Storz et al., 1990[62]; Demple, 1991[12]; Lushchak, 2001[40]). In *Escherichia coli*, an adaptive response to oxidative stress induced by O_2_^•^^−^ was shown to be coordinated by two differently transcribed genes, soxR and soxS, that operate in concert (Wu and Weiss, 1991[65]; Amábile-Cuevas and Demple, 1991[1]; Demple, 1991[12]). The oxidative signal is sensed by a [4Fe-4S] cluster in the SoxR protein, a member of the SoxRS regulon, which further transmits the signal to the transcriptional machinery resulting in *de novo* biosynthesis of various proteins including antioxidant enzymes (Demple, 1991[12]; Gaudu et al., 1997[20]; Lushchak, 2001[40]). The other regulatory pathway senses hydrogen peroxide and is called the OxyR regulon due to the key role of the OxyR regulatory protein that was first described in *Salmonella typhimurium* (Christman et al., 1985[8]) and later in *E. coli* (Christman et al., 1989[9]). Certain thiol groups of the OxyR protein were shown to sense oxidative signals providing a molecular mechanism to coordinate the bacterial adaptive response to H_2_O_2 _insults (Storz et al., 1990[62]; Lushchak, 2001[40], 2011[34]). Figure 3(Fig. 3) demonstrates the reactions in which the thiol groups of cysteine residues in the sensor proteins may participate. The cysteine residue is oxidized to a sulfenic derivative which can then be stabilized by the formation of intra- or intermolecular disulfide bonds. The oxidized sensor protein then up-regulates the transcription of target genes (e.g. those encoding antioxidant enzymes) either directly (as in the bacterial OxyR regulon) or indirectly (as in most other cases). However, in the early 2000's it was determined that earlier data on the high specificity of the bacterial regulon SoxRS to O_2_^•^^−^ were not accurate and showed that H_2_O_2 _could also up-regulate some enzymes of the SoxRS regulon. This was supported at two levels: (1) specific mRNA transcript levels of SoxR regulon genes (Manchado et al., 2000[42]; Zheng et al., 2001[66]) and (2) activities of the enzymes of the SoxRS regulon (Semchyshyn et al., 2005[53][54]). These findings clearly demonstrate tight connectivity and interplay between different regulatory pathways involved in the adaptive response to ROS insults.

Recently P. Jones and H. Sies (2015[30]) proposed the concept of a redox code that determines principles defining the positioning of the nicotinamide adenine dinucleotide (NAD, NADP), thiol/disulfide and other redox systems along with the thiol redox proteome spatio-temporal coordinates. Reversible oxidation of thiol groups plays a central role in the concept. Exposure of cysteine residues of sensor proteins to ROS results in their oxidation with the formation of sulfenic, sulfinic, or sulfonic acid derivatives (Figure 3(Fig. 3)). Sulfenic acid derivatives may be returned to their initial form by several reductases such as thioredoxin and glutaredoxin (Lushchak, 2012[38]). For a long time it was thought that sulfinic derivatives could not undergo reduction in biological milieu, but the discovery of sulfiredoxin (Biteau et al., 2003[5]; Findlay et al., 2005[15]; Neumann et al., 2009[44]) challenged this opinion. However, it is still believed that the sulfonic acid derivative of a cysteine residue cannot be reduced in living organisms. Due to these chemical transitions, reversible cysteine oxidation to sulfenic acid may be a useful marker for ROS sensing and could play a positive role in cell adaptation to oxidative insults. 

In addition to direct enzymatic reduction of sulfenic derivatives, formation of mixed thiols (e.g. by conjugating with reduced glutathione) is another way to prevent further oxidation of cysteine residues. For example, formation of such mixed thiols can prevent further oxidation of active thiol groups in proteins. Clearly, the protein in this form is not active, but it may be reduced again to return to its active form by interacting with a second glutathione molecule (Figure 3(Fig. 3)). Such processes result in restoration of an active sensor molecule and formation of an oxidized glutathione molecule that may be reduced by glutathione reductase (GR), usually at the expense of NADPH (Lushchak, 2014[37]). Finally, of NADPH levels are maintained by several NADP(H)-linked dehydrogenases with a pivotal role of glucose-6-phosphate dehydrogenase (G6PDH) which connects the antioxidant system with carbohydrate metabolism.

It should be noted that a similar redox chemistry as discussed above for the characterization of ROS sensing may also be applied for sensing one of the reactive nitrogen species (RNS) - nitric oxide (^•^NO). In bacteria, ^•^NO is believed to be sensed by the [4Fe-4S] cluster of the SoxR protein (Demple, 1991[12]; Lushchak, 2001[40]). In yeast we found that ^•^NO was sensed by Yap1 protein probably via formation of the S-nitroso derivative of the sensor protein (Lushchak et al., 2010[32]). The remaining elements coordinating the responses of yeast to nitrosative stress are virtually the same as those involved in their response to oxidative stress.

Interestingly, ROS sensing by regulatory pathways that are based on [Fe-S] clusters has been found only in bacteria, but not in eukaryotes to date. Such clusters occur in diverse eukaryote proteins like hydratases (aconitase, fumarase), but these are not members of any pathway known to regulate gene expression. Iron regulatory protein 1 may be the only exception found to date (Paraskeva and Hentze, 1996[47]; Volz, 2008[64]; Lushchak et al., 2014[33]). However, thiol-based signaling, but not [Fe-S]-based signaling, is widely used in all phylogenetic groups: bacteria, fungi, plants and animals. Interested readers are directed to several reviews in this field (Lushchak, 2010[41], 2011[34]; Flint et al., 2016[16]; Noctor et al., 2016[46]). In animals, the response to mild oxidative stress is coordinated by the ROS-sensitive Nrf2/Keap1 system that is based on the oxidation or electrophylic modification of cysteine residue/s of the sensor protein Keap1 (Nguyen et al., 2009[45]; Lushchak, 2012[38], 2014[37]; Baxter and Hardingham, 2016[3]; Sies, 2017[56]). 

## Conclusions and Perspectives

Reactive oxygen species are inevitable participants of vital processes. Their steady-state levels may be enhanced due to influences of various external or internal factors. In turn, this may cause certain physiological changes resulting in the development of oxidative stress. Great interest in ROS and oxidative stress from both basic and applied areas of biology/ biochemistry stimulated development of this field. In turn, this called for improvement of the definitions and formulations of the oxidative stress concept. Molecular mechanisms of organismal responses to oxidative stress have been disclosed over the course of the last 30 years and, in most cases, reversible oxidation of cysteine residues of sensory proteins was found to play a central role. 

However, although significant progress has been made in the field of oxidative stress, many problems remain to be resolved. Two critically important issues are: (1) development of reliable methods to monitor ROS levels *in vivo*, and (2) deciphering of details of the regulatory responses to oxidative insults by living organisms. The possibility to decrease the negative effects of ROS on organisms may be a good preventive or prophylactic strategy for many diseases where ROS are involved. This needs further investigation of the molecular mechanisms responsible for adaptive responses to oxidative insults and understanding where the border is between the possibility that an organism can provide an adequate response and recover from the oxidative stress and the impossibility of the organism to cope with the stress. Understanding such mechanisms will provide a tool to manage ROS-associated processes. A new emerging direction is investigation of the coordination of adaptive responses to oxidative stress under different physiological states. For example, this response depends on the age of an organism and may be even absent in old organisms (Pomatto and Davies, 2017[49]). Interaction between an organism's response to oxidative stress and its energetic status (Garaschuk et al., 2018[19]; Lushchak, 2021[39]) is another understudied field that promises to disclose molecular mechanisms of system responses to the stress. 

## Acknowledgement

The author (VIL) thanks Professor H. Sies who, sharing his knowledge, supported and stimulated VIL's interest in the field of oxidative stress. U. Dzaman and J.M. Storey are acknowledged for critical reading of the manuscript and U. Stambulska and N. Mosiichuk are acknowledged for assistance with manuscript and figure preparation. Hanse-Wissenschaftskolleg (Delmenhorst, Germany) is acknowledged for the excellent scientific environment which stimulated VIL to formulate intensity-based classification of oxidative stresses. VIL and KBS acknowledge grants from the Ministry of Education and Science of Ukraine and NSERC Canada that allowed development of our ideas via experimental studies on multiple model systems.

## Conflict of interest

The authors declare that they have no known conflict of interest.
